# Three-step fluoroscopy-guided percutaneous lumbar pedicle screw placement: a pilot study on technical feasibility, safety, and fluoroscopy reduction

**DOI:** 10.3389/fsurg.2026.1719831

**Published:** 2026-03-09

**Authors:** Wei Wu, Ke Jia, Fanguo Kong, Chao Chen

**Affiliations:** 1 Department of Orthopaedics, Union Hospital, Tongji Medical College, Huazhong University of Science and Technology, Wuhan, China; 2 Minimally Invasive Spinal Surgery Center, Luoyang Orthopedic-Traumatological Hospital of Henan Province (Henan Provincial Orthopedic Hospital), Zhengzhou, China

**Keywords:** lumbar disc herniated, lumbar spinal, lumbar spondylolisthesis, percutaneous pedicle screw placement, pilot study

## Abstract

**Background:**

Minimally invasive surgery has gained widespread popularity in clinical practice. Among spinal surgeries, percutaneous pedicle screw placement (PPS) is one of the most widely performed procedures. However, it necessitates high-frequency fluoroscopic guidance to ensure accuracy, resulting in substantial radiation exposure. Therefore, it is crucial to develop a technique that is fast, safe, and minimizes radiation exposure.

**Objective:**

We aimed to describe a novel three-step fluoroscopy-guided technique for PPS and preliminarily evaluate the technical feasibility, procedural efficiency, and short-term safety.

**Methods:**

This study prospectively enrolled consecutive patients who underwent PPS using either the three-step fluoroscopy-guided technique or the conventional method from December 2024 to February 2025, while data analysis was performed retrospectively. Data collected included operative time, fluoroscopy frequency, screw placement accuracy, and postoperative complications.

**Results:**

The three-step fluoroscopy-guided technique required an average of 4 ± 1.12 fluoroscopic exposures per screw, with an average screw placement time of 5.05 ± 0.923 min. In contrast, the conventional method required an average of 18.33 ± 2.89 fluoroscopic exposures per screw and an average placement time of 15.84 ± 4.11 min. And no significant complications, such as neural or vascular injuries, were reported.

**Conclusion:**

This pilot study suggests that the three-step fluoroscopy-guided PPS technique is technically feasible and may reduce fluoroscopy usage while maintaining short-term procedural safety, making it a feasible and efficient alternative that warrants further validation in larger cohorts.

## 1 Introduction

In recent decades, minimally invasive spine surgery has gradually gained prominence within the field of spinal surgery (1, 2). Among these techniques, percutaneous pedicle screw placement (PPS) is widely employed for the treatment of lumbar degenerative diseases (e.g., disc herniation, spinal stenosis), spinal fractures, and spinal instability (3–5). Compared with traditional open surgery, PPS can reduce surgical trauma, minimize postoperative complications, and accelerate patient recovery (6, 7). However, the successful execution of PPS relies heavily on intraoperative lumbar fluoroscopy to ensure precise positioning of pedicle screws. This dependency not only exposes both the patient and surgeon to radiation but also extends the operating duration. Prior fluoroscopy-guided PPS studies have reported wide variation in fluoroscopy utilization, ranging from approximately 5–6 shots per screw in single-AP-shot workflows to more than 10–20 shots per screw in conventional fluoroscopic workflows, depending on the technique and imaging protocol. Similarly, screw insertion time in fluoroscopy-guided PPS has been reported at around 2.6–3.0 min per screw in conventional K-wire-based workflows (3, 8).

Efforts to enhance the safety of pedicle screw insertion while minimizing the risk of cortical breach have led to the exploration of guiding instruments that preferentially advance along the cancellous channel of the pedicle. Among these, Watanabe et al. described the ball-tip technique for thoracic pedicle screw placement, in which a flexible probe with a rounded tip is advanced after initial cortical penetration to create a pilot channel. Owing to the difference in mechanical resistance between cancellous bone and the surrounding cortical shell, the probe tends to deflect along the inner cortical surface rather than penetrate it, a mechanism that has been shown to improve screw placement accuracy and reduce pedicle wall violation in both cadaveric and clinical studies (9). Nevertheless, the ball-tip technique was developed for open posterior spinal surgery and depends on direct exposure of the posterior elements, which limits its applicability to minimally invasive or PPS procedures. In percutaneous settings, repeated fluoroscopic confirmation during needle advancement remains a major source of radiation exposure and procedural inefficiency. Accordingly, there is a clear clinical need to adapt the principle of cancellous bone–guided advancement to a workflow compatible with percutaneous pedicle screw placement. To address the challenge, we developed a highly effective and safe percutaneous screw placement technique, termed the “Three-Step PPS.” This method leverages fluoroscopic imaging and the anatomical characteristics of the lumbar vertebrae, utilizing the controllability of a blunt-tipped guidewire within the cancellous bone of the pedicle to eliminate the need for repeated fluoroscopic verification for accurate needle insertion. This technique facilitates the rapid placement of unilateral pedicle screws while maintaining accuracy and safety.

Here, we systematically describe the operational procedure of the “Three-Step PPS,” evaluate its clinical value and analyze its unique advantages in reducing surgical complexity, improving the efficiency of lumbar pedicle screw placement, and ensuring safety. Our aim was to present a feasible and widely applicable percutaneous lumbar pedicle screwing technique for spine surgery.

## 2 Materials and method

### 2.1 Conventional PPS procedure

In the conventional PPS group, pedicle screw insertion was performed using the standard fluoroscopy-guided technique. After skin incision and soft tissue dilation, a Jamshidi needle was advanced toward the pedicle entry point under continuous anteroposterior and lateral fluoroscopic guidance. Repeated fluoroscopic confirmation was used to adjust the trajectory and depth of needle advancement until the pedicle was cannulated. A guidewire was then inserted through the Jamshidi needle, followed by sequential dilation and cannulated pedicle screw placement. Intermittent fluoroscopic imaging was performed throughout the procedure to confirm the trajectory, depth, and final position of the screws.

### 2.2 Surgical procedure

#### 2.2.1 Positioning the target lumbar segment

**Figure 1:**
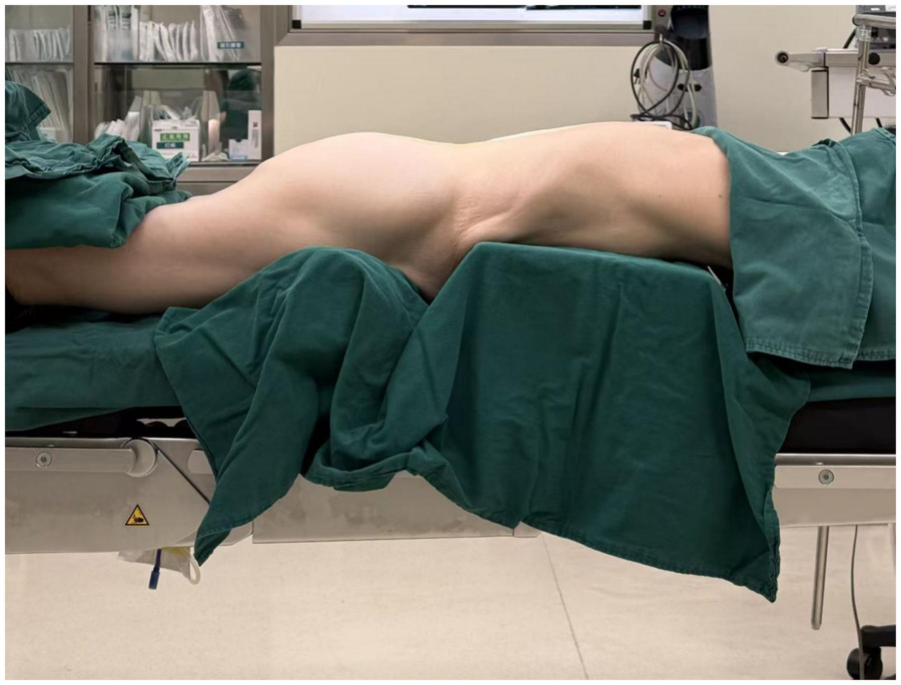
The patient is positioned prone, with the heights of the chest and hip supports adjusted to ensure the surgical lumbar segment is as horizontal as possible. This positioning facilitates accurate pedicle screw placement during the procedure.

Following the induction of satisfactory general anesthesia by the anesthesiologist, the patient is positioned prone. Particular attention is given to adjusting the height of the chest and hip supports to ensure that the target lumbar segment is as horizontal as possible, which is essential for improving the accuracy of the procedure (Figure 1).

#### 2.2.2 First fluoroscopy for identifying the entry point

**Figure 2:**
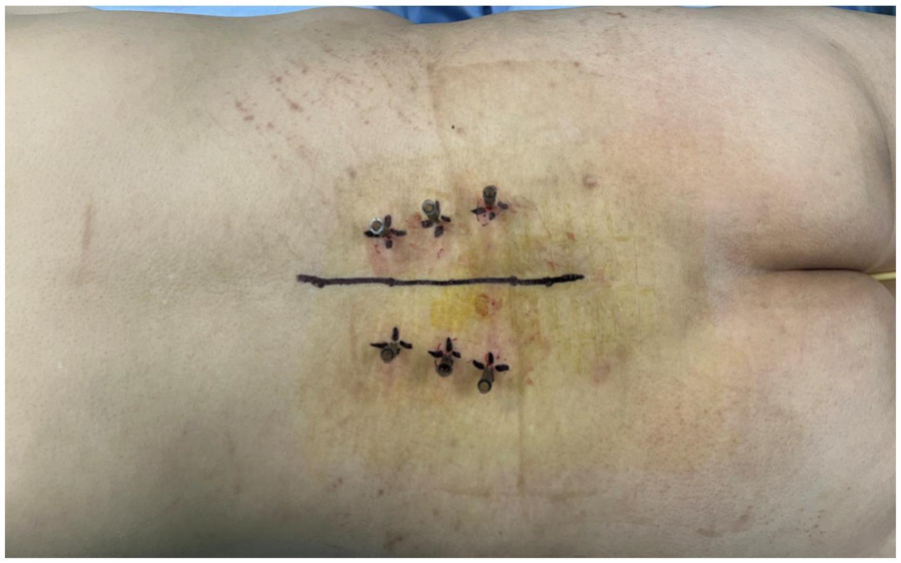
After skin disinfection, a sterile syringe needle is used for precise localization.

Following the disinfection of the operative area, a sterile syringe needle is used to mark and position the lumbar vertebral segments (Figure 2).

**Figure 3:**
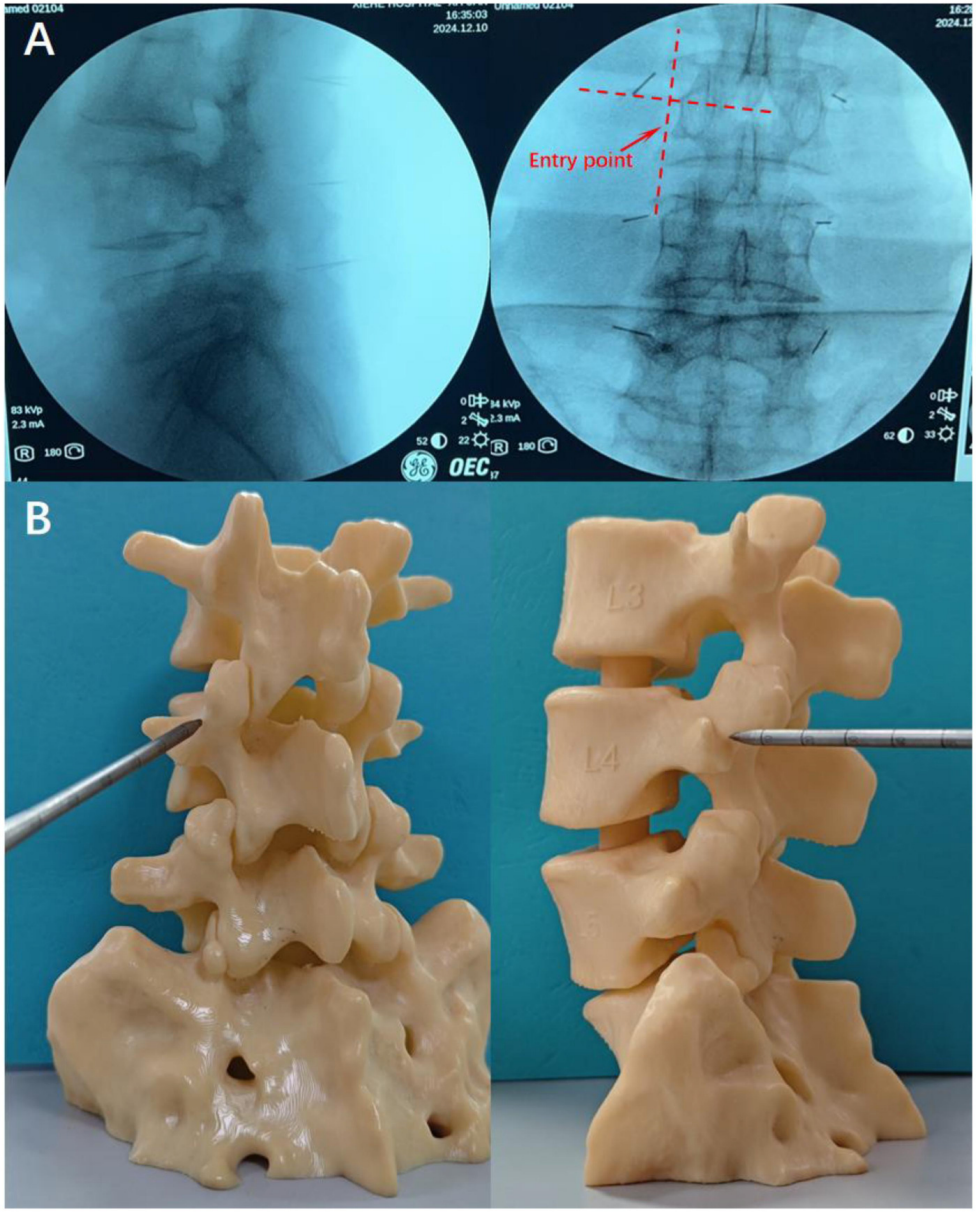
**(A)** The entry point is determined under fluoroscopy. In the anteroposterior view, the transverse process and articular process should be clearly visible. The entry point is located 1–2 mm below the intersection of the upper edge of the transverse process and the lateral edge of the superior articular process (red arrow). In the lateral view, the needle should align parallel to the upper part of the pedicle. **(B)** A 3D model illustrating the entry point.

Subsequently, the first fluoroscopic image is obtained using a C-arm x-ray to confirm the accuracy of the markings. The optimal fluoroscopic image should distinctly display the transverse process and articular processes in the anteroposterior view, with the needle tip aligned parallel to the upper portion of the pedicle in the lateral view. The final entry point should be located 1–2 mm below the intersection of the superior border of the transverse process and the lateral border of the superior articular eminence, ensuring an accurate trajectory for subsequent screw placement (Figure 3).

#### 2.2.3 Puncture at the entry point

Following routine area disinfection and sterile draping, the puncture trocar needle is positioned at the pre-identified entry point.

**Figure 4:**
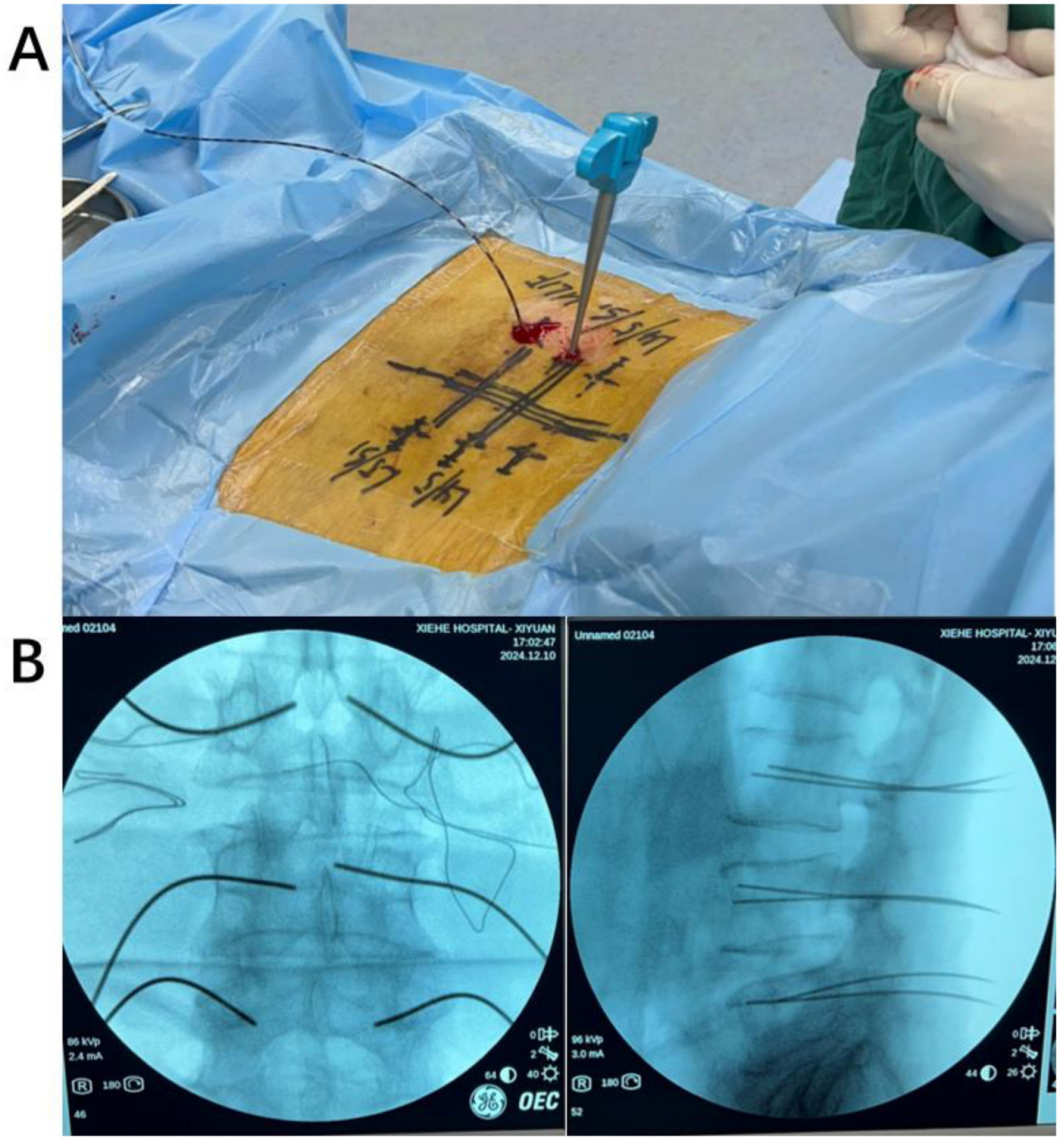
**(A)** After the surgeon breaches the cortical bone, the needle insertion is immediately halted, and a blunt-tip guidewire is introduced. **(B)** Second fluoroscopic imaging performed to confirm appropriate guidewire positioning.

The needle is then advanced along the trajectory corresponding to the intersection of the upper border of the transverse process and the outer margin of the superior articular process. Upon reaching this intersection, the needle is lowered by 1–2 mm and subsequently advanced slowly until it penetrates the cortical bone (Figure 4A).

#### 2.2.4 Insertion of the blunt-tip guidewire through the pedicle

After removal of the core of the puncture trocar needle, a 1.4 mm × 600 mm commercially available blunt-tip nickel–titanium guidewire is inserted into the trocar. Guidewire advancement does not rely solely on penetrating capability; instead, it is performed under tactile feedback by exploiting the difference in mechanical resistance between cancellous bone and cortical bone, allowing the guidewire to deflect along the inner cortical surface rather than breach the cortex. After guidewire advancement, a second fluoroscopic examination is performed using a C-arm x-ray system to confirm accurate positioning (Figure 4B).

#### 2.2.5 Placement of the lumbar pedicle screw

**Figure 5:**
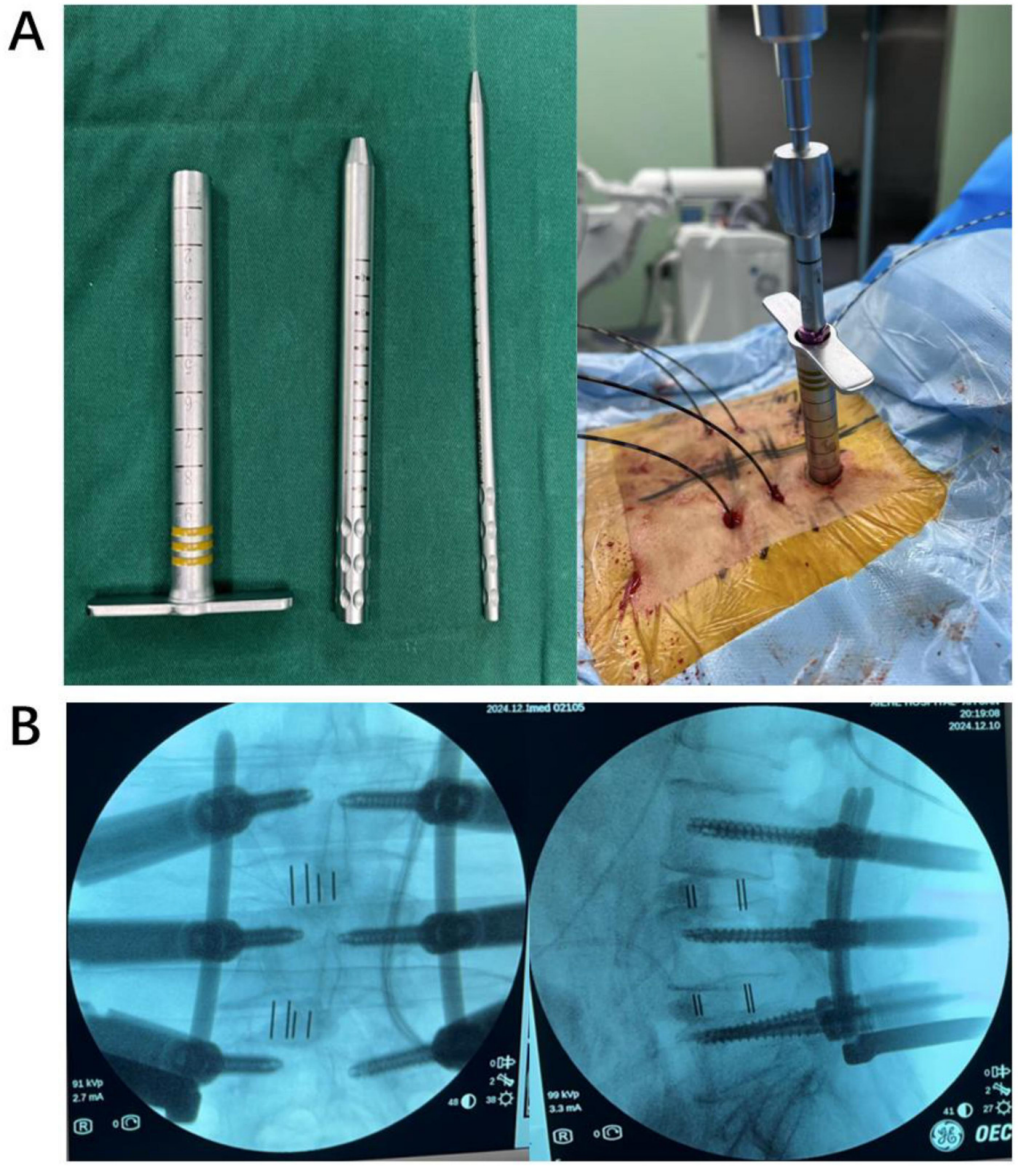
**(A)** The left panel demonstrates the sequential dilator tubes. Based on the guidewire’s position, sequential dilators are used to expand the screw path, followed by the insertion of the pedicle screw. **(B)** After the pedicle screw is inserted, a third fluoroscopic imaging is performed to confirm proper placement.

**Figure 6:**
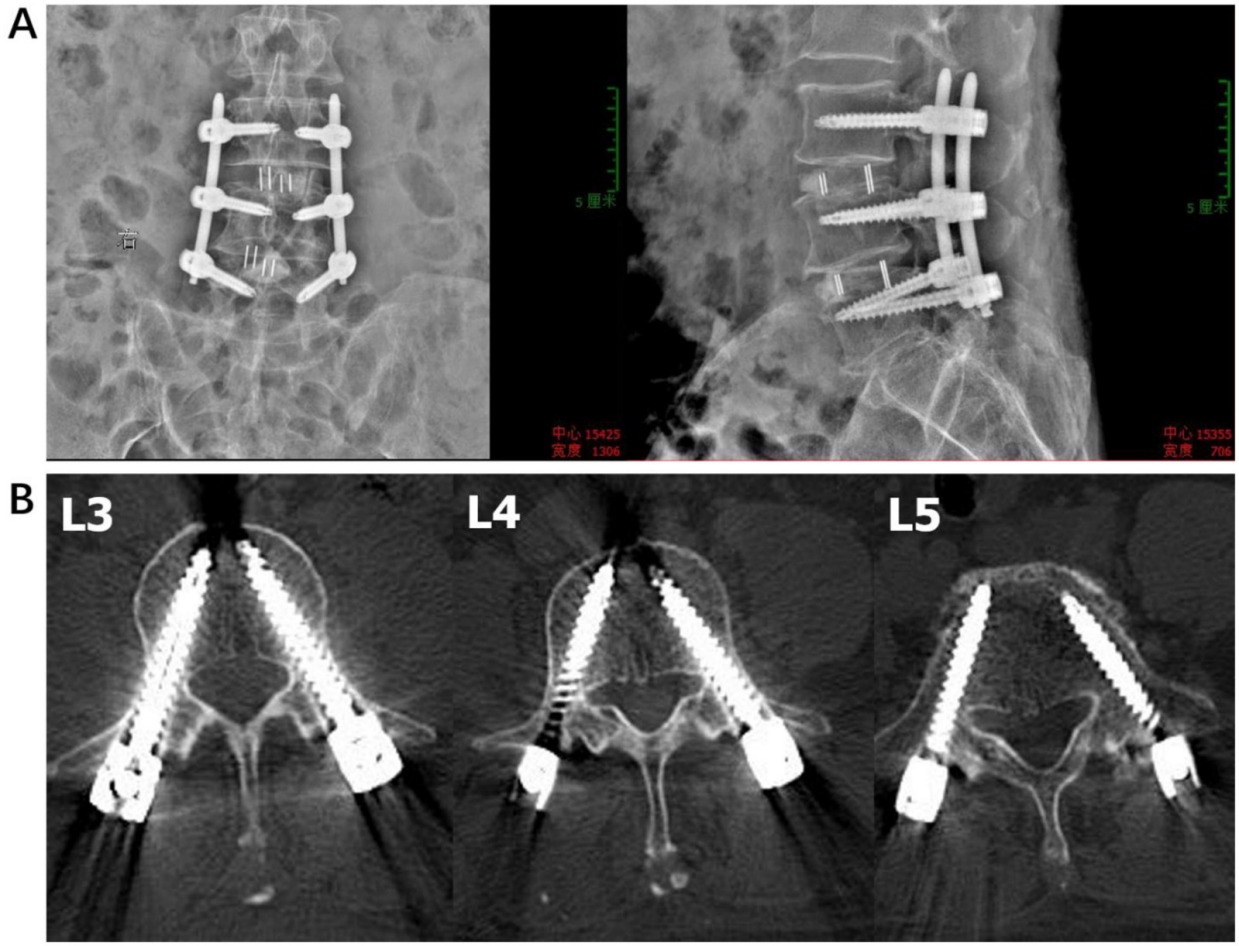
Postoperative imaging of a patient with lumbar disc herniation undergoing three-step fluoroscopic guided PPS surgery. **(A)** Anteroposterior and lateral x-ray views. **(B)** CT scan of the L3-L5 vertebrae, demonstrating satisfactory placement of the pedicle screws.

Based on the surgical requirements, an appropriately sized lumbar pedicle screw is selected. Sequential dilators are used along the guidewire path to enlarge the screw tract, ensuring smooth and safe screw insertion. The pedicle screw is then inserted into the vertebral body (Figure 5A). Following screw placement, a third fluoroscopic examination is performed using a C-arm x-ray machine to confirm proper positioning of the pedicle screws. Upon satisfactory confirmation, the procedure is considered complete (Figure 5B). Here, we present two postoperative images illustrating pedicle screws placed using the “Three-Step PPS” technique (Figure 6). longer follow-up are warranted to further validate these preliminary results.

### 2.3 Inclusion and exclusion criteria

**Inclusion criteria: A.** Patients who underwent PPS for lumbar degenerative disease, lumbar spine fractures, or other indications. **B.** Their surgeries were performed by the authors' surgical team. **C.** They had complete clinical records, including preoperative and postoperative imaging as well as follow-up data. **D.**

They underwent either the conventional PPS technique or the “Three-Step PPS” technique. **E.** They were aged 18 years or older to ensure skeletal maturity and data consistency (10).

**Exclusion criteria: A.** Patients with altered pedicle anatomy or significant anatomical abnormalities in the surgical area (e.g., severe lumbar spondylolisthesis or pronounced scoliosis) **B.** Patients who declined to undergo surgical treatment. **C.** Patients with concomitant systemic illnesses. **D.** Patients with incomplete follow-up data. **E.** Cases involving non-technical intraoperative complications, such as surgery being aborted due to unforeseen circumstances or converted to another surgical technique.

### 2.4 Patient information

Between December 2024 and February 2025, we prospectively enrolled 24 patients undergoing pedicle screw placement. Among them, 12 patients received the conventional PPS technique (PPS group), while the remaining 12 were treated using the “Three-Step PPS” method (“Three-Step PPS” group). Baseline characteristics of the patients are summarized in Table 1. The PPS group comprised five male and seven female patients, with an average age of 60.08 years, while the “Three-Step Fluoroscopy-Guided PPS” group comprised six male and six female patients, with an average age of 56.17 years. Most patients in both groups were diagnosed with lumbar disc herniation, lumbar spinal stenosis, or lumbar spondylolisthesis, with a few cases also presenting comorbidities such as hypertension, diabetes, and scoliosis. The most commonly affected lumbar segment in both groups was L4/5. Additionally, no significant differences were observed between the two groups in terms of disease severity, as indicated by similar Visual Analogue Scale (VAS) scores (5.33 ± 2.06 vs. 5.08 ± 1.73, *p* = 0.751) and Oswestry Disability Index (ODI) scores (51.92 ± 11.45 vs. 44.5 ± 10.37, *p* = 0.11).

**Table 1:** Baseline information for patients who underwent conventional PPS or “three-step PPS” technique.

Case	Gender	Age	Diagnose	Surgery	Spine levels operated	BMI (kg/m^2^)	BMD (g/cm^2^)	Underlying disease	Duration of symptoms (months)	VAS	ODI
1	Male	68	Lumbar spondylolisthesis; lumbar spinal stenosis	“three-step”PPS	L4/5	19.2	1.03	/	0.1	3	42
2	Male	64	Lumbar spondylolisthesis; lumbar spinal stenosis	“three-step”PPS	L4/5	21.5	1.09	/	48	3	33
3	Male	24	Herniated lumbar disk; lumbar spinal stenosis	“three-step”PPS	L4/5; L5/S1	17.5	0.96	/	0.07	6	51
4	Male	66	Herniated lumbar disk; lumbar spondylolisthesis	“three-step”PPS	L4/5	25.1	1.05	Hypertension	6	5	56
5	Female	54	Herniated lumbar disk; lumbar spondylolisthesis	“three-step”PPS	L4/5; L5/S1	21	1.08	/	48	8	78
6	Male	73	Lumbar spinal stenosis; lumbar spondylolisthesis	“three-step”PPS	L4/5	19.8	1	Hypertension; diabetes	3	3	38
7	Female	62	Herniated lumbar disk; lumbar spinal stenosis	“three-step”PPS	L4/5	21.7	1.04	/	8	3	50
8	Female	72	Lumbar spinal stenosis; Lumbar compression fracture	“three-step”PPS	L4/5	20.9	1.12	/	12	5	58
9	Female	54	Lumbar spinal stenosis; lumbar spondylolisthesis	“three-step”PPS	L4/5	23.4	0.98	Scoliosis	24	5	72
10	Female	63	Lumbar spinal stenosis	“three-step”PPS	L3/4	20.6	1.06	Scoliosis	1	8	91
11	Female	60	Lumbar spinal stenosis; lumbar spondylolisthesis	“three-step”PPS	L4/5	19.6	1.02	/	24	7	62
12	Female	61	Lumbar spinal stenosis	“three-step”PPS	L4/5	21.3	1.1	/	12	8	70
13	Female	56	Lumbar spinal stenosis; lumbar spondylolisthesis	PPS	L4/5	22.6	1.07	/	6	4	28
14	Female	59	Herniated lumbar disk; lumbar spondylolisthesis; lumbar spinal stenosis	PPS	L4/5; L5/S1	18.4	0.99	/	60	5	38
15	Female	65	Herniated lumbar disk; lumbar spinal stenosis	PPS	L4/5	20.6	1.01	/	0.05	3	25
16	Female	52	Herniated lumbar disk; lumbar spondylolisthesis; lumbar spinal stenosis	PPS	L3/4	21.9	1.08	Hypertension	12	9	95
17	Female	74	Lumbar spondylolisthesis; lumbar spinal stenosis	PPS	L4/5	26.1	1.11	/	6	2	42
18	Female	35	lumbar spondylolisthesis; lumbar spinal stenosis	PPS	L3/4	19.7	1.03	Scoliosis	12	5	45
19	Male	65	Lumbar spinal stenosis	PPS	L3/4; L4/5	21.2	1.02	/	36	5	58
20	Male	24	Herniated lumbar disk; lumbar spinal stenosis	PPS	L4/5; L5/S1	20.8	1.06	/	24	6	60
21	Male	52	Herniated lumbar disk; lumbar spinal stenosis	PPS	L4/5; L5/S1	24.2	1.04	/	1	6	72
22	Male	54	Lumbar spondylolisthesis; lumbar spinal stenosis	PPS	L4/5	20.5	0.97	Scoliosis	48	6	52
23	Male	74	Herniated lumbar disk; lumbar spinal stenosis	PPS	L4/5	19.5	1	/	0.008	5	42
24	Male	64	Herniated lumbar disk; lumbar spinal stenosis	PPS	L4/5	21.2	1.1	Scoliosis	24	5	35

### 2.5 Postoperative CT evaluation and blinding

Postoperative computed tomography (CT) scans were independently reviewed by two experienced spine surgeons who were blinded to the surgical technique and group allocation. Pedicle screw placement accuracy was evaluated using a standardized grading system. Any discrepancies between reviewers were resolved through consensus discussion.

## 3 Results

To evaluate the potential advantages of the “Three-Step PPS” technique, we compared multiple operative parameters between the two surgical approaches, including pedicle screw placement accuracy, operative duration, intraoperative fluoroscopy frequency, and overall safety. All procedures were performed by the same surgical team to minimize operator-related variability and reduce procedural bias. The key information is summarized in Table 2.

**Table 2:** Outcome evaluation for patients receiving PPS or “three-step PPS” technique.

Variable	“Three-step”PPS	PPS	Effect size (*t*/*X*^2^/*Z*)	*p* value
Cases (No.)	12	12		
Gender (male/female)	6/6	5/7	*X*^2^ = 0.168	*p* > 0.5
Age (Mean ± SD, years)	59.77 ± 12.03	57.27 ± 15.03	t = 0.44	*p* = 0.664
Duration of symptoms (months)	14.97 ± 18.98	19.59 ± 20.44	t = 0.57	*p* = 0.58
Accuracy of screw implantation (%)				
Grade 0	11/12	11/12	*Χ*^2^ = 0	*p* = 1
Grade 1	0	1/12		
Grade 2	1/12	0		
Grade 3	0	0		
Grade 4	0	0		
Operating time/numbers of screws (min)	5.05 ± 0.923	15.84 ± 4.11	*t* = −6.41	*p* < 0.001
Number of fluoroscopes (times)	4 ± 1.12	18.33 ± 2.89	*t* = −14.89	*p* < 0.001
Nerve damage within one week after surgery (cases)	0	1		*p* = 1
Wound infection (cases)	0	2		*p* = 0.478
Postoperative hematoma (cases)	0	0		
Pedicle screw malposition (cases)	0	0		
Postoperative 3-month VAS score	5.33 ± 2.06	5.08 ± 1.73	0.322	*p* = 0.751
Postoperative 3-month ODI score	58.42 ± 17.16	49.33 ± 19.78	1.2	*p* = 0.242

### 3.1 Pedicle screw placement accuracy

The accuracy of the placed pedicle screws was assessed using postoperative CT imaging. Cases were assessed based on a previously established grading system: Grade 0 indicates no pedicle breach; Grade 1 indicates a breach of less than 2 mm; Grade 2 indicates a breach between 2 and 4 mm; Grade 3 indicates a breach between 4 and 6 mm; and Grade 4 indicates complete displacement of the screw outside the pedicle (Table 3) (11–13). This classification enabled a direct comparison of accuracy between the “Three-Step PPS” group and the PPS group. According to our results, in the “Three-Step PPS” group, only one case was classified as Grade 1, while all others were Grade 0. In the conventional PPS group, only one case was classified as Grade 2, with the remaining cases also graded as 0. Therefore, both groups demonstrated high accuracy, with no significant difference between them (*p* > 0.05).

**Table 3:** Pedicle screw breach grade distribution.

Grade	Screw position
0	No pedicle breach
1	Breach <2 mm
2	Breach 2–4 mm
3	Breach 4–6 mm
4	Complete displacement

### 3.2 Screw placement time and fluoroscopy frequency

Our retrospective data analysis of screw placement time and intraoperative fluoroscopy frequency in these 24 prospectively enrolled cases revealed that the operative time and intraoperative fluoroscopy frequency in the “Three-Step PPS” group were significantly lower than those in the PPS group (*p* < 0.001). Specifically, the average placement time per screw was 5.05 ± 0.923 min in the “Three-Step PPS” group, compared to 15.84 ± 4.11 min in the PPS group. Additionally, the average fluoroscopy frequency was 4 ± 1.12 instances in the “Three-Step PPS” group, vs. 18.33 ± 2.89 instances in the conventional PPS group. These findings highlight the potential of the “Three-Step PPS” technique to enhance surgical efficiency and reduce radiation exposure by decreasing both intraoperative fluoroscopy instances and surgical time.

### 3.3 Safety

Our results indicate that the “Three-Step PPS” technique demonstrated comparable short-term safety compared to conventional PPS, with no increase in observed complications. No postoperative complications were observed in the “Three-Step PPS” group, whereas one case of nerve injury was recorded in the PPS group on postoperative day 2. Although the incidence of nerve injury was low in both groups, the nerve injury in the conventional PPS group highlights a potential risk of postoperative complications that may require additional treatment. In contrast, the absence of such complications in the “Three-Step PPS” group suggests that short-term safety was maintained without an observed increase in complications.

Overall, the “Three-Step PPS” technique demonstrates improved surgical efficiency and reduced fluoroscopy use while maintaining comparable short-term safety. Notably, by effectively minimizing radiation exposure, the “Three-Step PPS” technique offers a safer and more efficient surgical option.

## 4 Discussion

The “Three-Step PPS” technique introduced in this study offers the distinct advantage of significantly reducing screw placement time. In the traditional percutaneous pedicle screw insertion method, the puncture needle typically features rigid, sharp tips, increasing the risk of breaching the inner or lower cortical wall of the pedicle, which could potentially result in spinal cord or nerve root injury (14, 15). This not only demands a high level of surgical skill from the surgeon but also requires repeated fluoroscopy to confirm the position of the needle, thereby increasing the complexity and difficulty of the procedure. In addition, the multiple fluoroscopies involved in the traditional method elevate the risk of radiation exposure to both patients and medical staff, while also prolonging the duration of the operation (16–18). Although advanced technologies such as computer navigation and robotic assistance have significantly improved the accuracy of lumbar pedicle screw placement, these techniques are often costly, complex, and reliant on equipment, limiting their widespread adoption (19–21). In contrast, the “Three-Step PPS” technique presents a practical and scalable alternative.

This technique innovatively incorporates a blunt-tip, high-strength nickel-titanium alloy guidewire, building upon the traditional puncture needle. The guidewire safely traverses the cancellous bone of the pedicle and vertebral body without damaging the cortical bone, thereby reducing reliance on puncture needles and minimizing the need for multiple fluoroscopic images, which are commonly required in traditional methods. This innovative design effectively improves safety by reducing radiation exposure through less frequent fluoroscopy. Additionally, the procedure time is significantly reduced owing to more efficient and straightforward intraoperative maneuvers. For example, our team can now place a single lumbar pedicle screw guidewire in under 1 min, achieving highly satisfactory screw placement. This advancement not only improves procedural efficiency but also greatly reduces the potential risks of intraoperative bleeding, infection, and prolonged anesthesia.

The advantages of this technique make it widely applicable in several clinical scenarios. In particular, it provides an efficient and safe solution for lumbar interbody fusion, lumbar fractures, and procedures requiring percutaneous localization, such as percutaneous kyphoplasty (PKP) and percutaneous vertebroplasty (PVP). This technique not only enhances convenience but also broadens the treatment options available to clinicians.

Although the “Three-Step PPS” technique demonstrates superior performance and safety in most cases, it does have certain limitations in specific situations. First, for patients with severe lumbar spondylolisthesis or scoliosis, determining a reliable entry point can be challenging due to altered anatomical structures and abnormal growth of the articular synchondrosis. In these cases, the procedure becomes more difficult, and the puncture guidewire may not penetrate smoothly. In addition, in younger patients, especially those with higher bone density, the blunt tip may struggle to penetrate the cancellous bone due to its increased hardness. In such instances, traditional rigid puncture needles remain a feasible alternative, as this method allows for flexible adaptation to conventional techniques when necessary. And we acknowledge that detailed pedicle-level morphometric parameters, such as the occupancy ratio at the narrowest part of the pedicle and quantitative assessment of cancellous bone sclerosis, were not included in this pilot study. These indices were not prospectively collected and could not be reliably reconstructed retrospectively due to the lack of standardized CT-based measurement protocols and potential measurement bias. As a complementary baseline indicator, bone mineral density (BMD) was therefore included to reflect overall bone quality. Future prospective studies incorporating standardized morphometric assessment may further refine anatomical stratification and validate the generalizability of this technique.In addition, although the present technique can significantly reduce operative time and fluoroscopy usage, some intraoperative adjustments and fluoroscopic confirmation may still be required for some patients with complex spinal deformities or anatomical abnormalities. Therefore, despite its advantages, the new technique should be applied with caution and flexibly adapted in combination with traditional approaches in certain special cases. Furthermore, this study should be interpreted as a pilot investigation primarily designed to assess technical feasibility and short-term procedural safety. Given the limited sample size and short observation period, the findings are not intended to provide definitive comparative clinical outcomes. In addition, the mixed prospective enrollment with retrospective data analysis design should be acknowledged as a methodological limitation. Larger, prospective studies with longer follow-up are warranted to further validate these preliminary results. In addition, quantitative radiation dose metrics such as mGy or DAP were not available, which represents a limitation of the present study.

Finally, the technique is currently limited to lumbar spine surgery, and its application has not yet been extended to the cervical or thoracic segments. In addition, our team has not utilized this technique for L1 and L2 lumbar segments, which may be a limitation of our approach. Several technical limitations of the present study should be acknowledged. The proposed three-step technique relies heavily on tactile feedback and the mechanical resistance difference between cancellous and cortical bone. Therefore, its applicability may be reduced in patients with severe spinal deformity, markedly sclerotic pedicles, or extremely high bone density, in whom guidewire advancement may encounter increased resistance and fluoroscopic guidance may still be required. In addition, a learning curve is inevitably associated with this technique. Based on our observational clinical experience rather than formal statistical modeling, procedural proficiency—characterized by stable tactile perception, controlled guidewire advancement, and reduced fluoroscopy usage—was generally achieved after approximately 10–15 cases, after which operative time and fluoroscopy exposure tended to plateau. This information may be valuable for surgeons during the early adoption phase. In the future, we aim to validate this technique in a broader patient population to further evaluate its indications and clinical outcomes, and “three-step”PPSwork towards further validation and potential broader implementation of the “Three-Step PPS” technique in clinical practice.

## Data Availability

The raw data supporting the conclusions of this article will be made available by the authors, without undue reservation.
